# A distance–performance trade‐off in the phenotypic basis of dispersal

**DOI:** 10.1002/ece3.5583

**Published:** 2019-08-22

**Authors:** Brett R. Addis, Bret W. Tobalske, Jon M. Davenport, Winsor H. Lowe

**Affiliations:** ^1^ Division of Biological Sciences University of Montana Missoula MT USA; ^2^ Department of Biology Appalachian State University Boone NC USA

**Keywords:** dispersal distance, locomotion, plethodontid salamanders, trade‐off

## Abstract

Across taxa, individuals vary in how far they disperse, with most individuals staying close to their origin and fewer dispersing long distances. Costs associated with dispersal (e.g., energy, risk) are widely believed to trade off with benefits (e.g., reduced competition, increased reproductive success) to influence dispersal propensity. However, this framework has not been applied to understand variation in dispersal distance, which is instead generally attributed to extrinsic environmental factors. We alternatively hypothesized that variation in dispersal distances results from trade‐offs associated with other aspects of locomotor performance. We tested this hypothesis in the stream salamander *Gyrinophilus porphyriticus* and found that salamanders that dispersed farther in the field had longer forelimbs but swam at slower velocities under experimental conditions. The reduced swimming performance of long‐distance dispersers likely results from drag imposed by longer forelimbs. Longer forelimbs may facilitate moving longer distances, but the proximate costs associated with reduced swimming performance may help to explain the rarity of long‐distance dispersal. The historical focus on environmental drivers of dispersal distances misses the importance of individual traits and associated trade‐offs among traits affecting locomotion.

## Graphical abstract

Variation in dispersal distance is generally attributed to extrinsic environmental factors. We alternatively hypothesized that variation in dispersal distance results from trade‐offs associated with other aspects of locomotor performance. We tested this hypothesis in the stream salamander *Gyrinophilus porphyriticus* and found that salamanders with long forelimbs dispersed farther in the field but swam at slower velocities under experimental conditions, indicating that a distance–performance trade‐off likely shapes selection on dispersal distances.

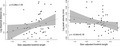

## 1 INTRODUCTION

Dispersal is a key driver of ecological and evolutionary processes by affecting population growth rates and gene flow (Tittler, Fahrig, & Villard, 2006; Van Houtan, Pimm, Halley, Bierregaard, & Lovejoy, 2007). Across taxa, most individuals in natural populations do not disperse, whereas dispersal distances vary substantially among those that do, with few individuals exhibiting long‐distance dispersal (Endler, 1977; Johnson & Gaines, 1990; Mayr, 1963). However, most dispersal research has focused on the discrete emigration response (stay vs. leave), leaving us with little understanding of the factors influencing individual variation in dispersal distance. Identifying the factors that underlie variation in dispersal distance is critical because long‐distance dispersal contributes disproportionately to range shifts (Higgins, Richardson, & Fahrig, 1999), invasions (Kot, Lewis, & Driessche, 1996; Lindström, Håkansson, & Wennergren, 2011; Miller & Tenhumberg, 2010), and population persistence (Bohrer, Nathan, & Volis, 2005).

Dispersal incurs energy, mortality risk, and opportunity costs that are widely believed to trade off with the benefits of dispersal (e.g., reduced competition, increased reproductive success) to influence the propensity to disperse (Bonte et al., 2012; Clobert, Galliard, Cote, Meylan, & Massot, 2009; Ronce & Clobert, 2012). We now have evidence from multiple taxa that dispersing individuals are not a random subset of the population and, instead, differ from residents in morphological, physiological, and behavioral traits (Benard & McCauley, 2008; Edelaar & Bolnick, 2012; Harrison, 1980). These differences may indicate selection for “dispersal phenotypes,” but phenotypic attributes that facilitate dispersal may also induce costs. For example, investment in flight structures for dispersal creates a resource allocation trade‐off with reproduction in many wing dimorphic insects (Denno, Olmstead, & McCloud, 1989; Mole & Zera, 1993). However, the influence of phenotypic variation across dispersing individuals on dispersal distances is relatively unstudied due to the difficulty of directly quantifying dispersal distances in the field (Koenig, Vuren, & Hooge, 1996; Lowe & McPeek, 2012; Nathan, 2001). The rarity of long‐distance dispersal alone suggests that it is costly, and highlights the need to consider the possibility that phenotypic specialization for long‐distance dispersal also creates costs that have gone unrecognized. Indeed, cost–benefit trade‐offs are well documented for the discrete emigration response, but this framework has not been applied to understand variation across individuals in dispersal distance. Instead, variation in dispersal distance is often attributed to extrinsic stochastic or environmental factors (Carlquist, 1981; Morales, 2002; Tufto, Engen, & Hindar, 1997), rather than phenotypic attributes of the individual.

Locomotor performance seems a likely candidate to influence distances that individuals move, as well as potential costs of long‐distance dispersal. Locomotion serves many different functions, including foraging, prey capture, predator escape, and dispersal, each requiring different morphological or physiological specializations. In aquatic vertebrates, morphological specialization to maximize stability and reduce drag comes at a cost to maneuverability (Webb, 1984; Weihs, 2002). These locomotor performance differences may allow sustained swimming for long‐distance dispersal, but create a cost through reduced fast‐starts for prey capture or predator escape. We cannot, however, assess such trade‐offs using indirect, proximate indices of dispersal ability (e.g., velocity, acceleration, maneuverability) because this common approach inherently confounds dispersal with other aspects of locomotor performance (Arnold, Cassey, & White, 2016; Bringloe, Drolet, Barbeau, Forbes, & Gerwing, 2013; Cormont et al., 2011). The lack of direct data on individual dispersal distances and their associated phenotypes under natural conditions has, until now, precluded more rigorous assessment.

**Figure 1:**
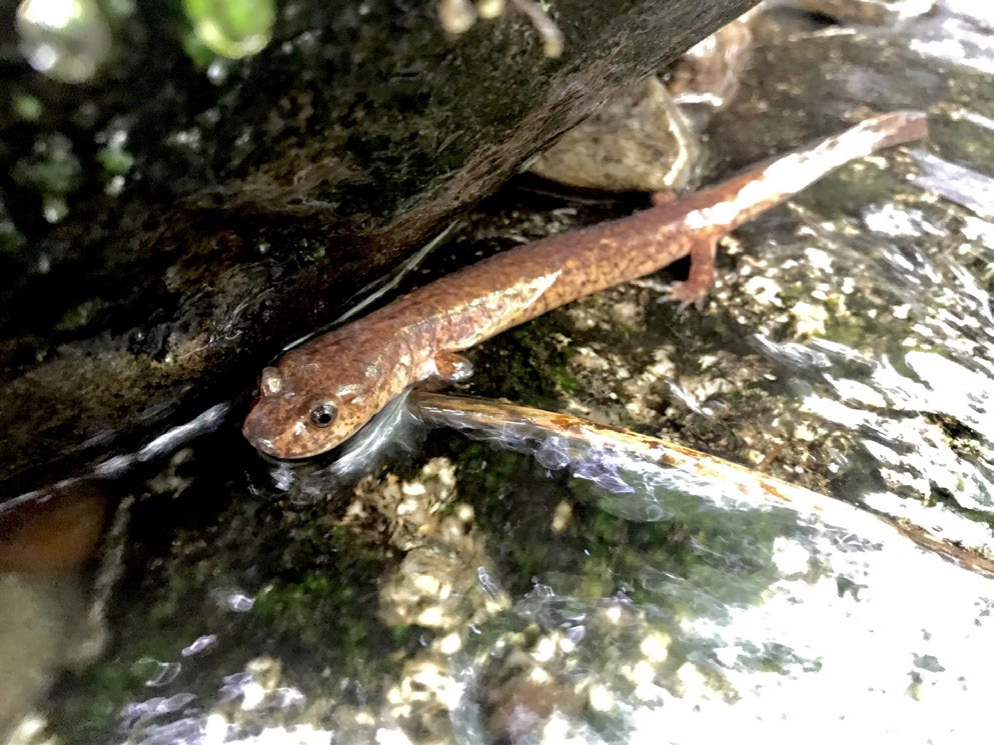
*Gyrinophilus porphyriticus* (photograph by Maddy Cochrane)

We assessed locomotion‐based trade‐offs associated with dispersal distance in the stream salamander *Gyrinophilus porphyriticus* (Figure 1). Our goal was to provide novel empirical insight on whether phenotypic attributes associated with variation in dispersal distance constrain other aspects of locomotor performance. First, we used 4 years of intensive, spatially explicit capture–mark–recapture data to test for a morphological basis of dispersal distance under natural field conditions. Trunk and leg morphology are known to affect swimming, underwater walking, and terrestrial walking performance in salamanders, respectively (Ashley‐Ross, Lundin, & Johnson, 2009; Azizi & Horton, 2004; D'Août & Aerts, 1997), leading to predictions that these traits may influence dispersal distance in *G. porphyriticus*. Next, we tested whether morphological traits related to dispersal distance in the field also influenced swimming performance in an experimental water chamber. *Gyrinophilus porphyriticus* larvae and adults may disperse by swimming, or, given the turbulent nature of headwater streams, may instead walk along the stream bottom or adults only may walk on land (Greene, Lowe, & Likens, 2008; Grover & Wilbur, 2002). However, regardless of the mode of locomotion employed for dispersal, swimming is likely important for other ecological functions, including capture of invertebrate prey and escape from aquatic predators (Brodie, Nowak, & Harvey, 1979; Petranka, 1988; Resetarits, 1995).

## 2 MATERIALS AND METHODS

### 2.1 Study species and site

*Gyrinophilus porphyriticus* belongs to the Plethodontidae, the lungless salamanders, and is found in small, cool, well‐oxygenated streams along the Appalachian uplift in the eastern United States (Petranka, 1988). Larvae are exclusively aquatic (Bruce, 1980) and adults are mainly aquatic but can forage terrestrially at night (Deban & Marks, 2002; Degraaf & Rudis, 1990). During the day, larvae and adults are found in interstitial spaces among cobble (Bruce, 2003). The larval period lasts 3–5 years (Bruce, 1980) and adults can live to be 14 years (W.H. Lowe, *unpublished data*). Previous work in this system has shown that both larval and adult *G. porphyriticus* disperse (Lowe, 2003; Lowe, Likens, & Cosentino, 2006), so both life stages were the focus of this study. This species is suited for dispersal studies because movements are generally constrained to linear stream corridors, so detection probability is less affected by movement distance, overcoming a major empirical hurdle (Koenig et al., 1996). Additionally, the relative mobility of *G. porphyriticus* is low, so surveys can detect a wide range of dispersal distances, including rare long‐distance dispersal events.

This work was conducted in three hydrologically independent first‐order streams (Bear, Paradise, Zigzag) in the Hubbard Brook Experimental Forest, located in the White Mountains of central New Hampshire (43°56′N, 71°45′W). These streams differ in environmental conditions, including aspect, daily discharge, and drainage slope (Lowe, Likens, McPeek, & Buso, 2006; McGuire et al., 2014).

### 2.2 Survey methods

Capture–mark–recapture surveys were conducted in June–September of 2012–2015. One‐kilometer sections encompassing the majority of the perennial portion of each stream were surveyed nine times throughout each summer, for a total of 36 surveys per stream over the 4‐year study period. A constant search effort was maintained by turning one cover object per meter of stream; thus, surveys provided spatially explicit information about the capture locations of individual salamanders. Previously unmarked salamanders were injected with visible implant elastomer (Northwest Marine Technologies). All encountered individuals were photographed for the purpose of quantifying morphology (see below), and snout–vent length (SVL) was recorded.

### 2.3 Quantifying dispersal distance

We quantified dispersal distances in recaptured individuals as the net distance moved (m along the stream) over the 4‐year study period. Due to the rarity of long‐distance dispersal, it was necessary to pool movement data across streams, sexes, life‐history stages, and time to achieve sufficient sample sizes to test for relationships between morphology, dispersal distance, and swimming performance. Previous surveys of *G. porphyriticus* showed no differences in movement distributions of adults versus larvae or males versus females (Lowe, 2003; Lowe, Likens, & Cosentino, 2006). Additionally, movement is not influenced by intraannual variation in stream flow (Lowe, 2003; Lowe, Likens, & Cosentino, 2006), justifying pooling movement data across streams.

Home ranges in *G. porphyriticus* are approximately 3 m^2^ (Lowe, 2003), which roughly translates to 3 m in stream length. Therefore, we considered a dispersal event as any movement >4 m in stream length from an initial location to ensure that dispersal movements were distinct from daily movements within the home range (Burgess, Baskett, Grosberg, Morgan, & Strathmann, 2015; Van Dyck & Baguette, 2005). There was a strong correlation between the total distance moved over the study period and net movement from the initial capture location in individuals that were recaptured more than once (*n* = 34, *r* = .86, *p* < .001), indicating that most dispersal movements are unidirectional and permanent.

### 2.4 Morphological analyses

To test whether individual variation in trunk and limb morphology was associated with differences in dispersal distance, we photographed each captured individual alongside a ruler and measured trunk width, trunk length, humerus length, and femur length from these digital photographs. Humerus length and femur length served as proxies for fore‐ and hindlimb morphologies, as obtaining accurate measurements of the distal portions of the limbs from photographs was generally not possible. Because we expected all body measurements to be correlated with the overall size of the animal (SVL), we generated size‐adjusted shape variables using principle component analysis (Adams & Beachy, 2001; Cosentino & Droney, 2016). We extracted two principal components from each of four covariance matrices representing the four body elements. Each covariance matrix included log‐transformed SVL and one of the four body measurements (log‐transformed). The first principal components (PC1) represented the generalized size of the salamander, and the second principal components (PC2) represented size‐adjusted morphological characters.

To test for an association between morphology and dispersal distance, we performed stepwise multiple regression analysis to identify size‐adjusted morphological characters (PC2s) that best predicted dispersal distance in individuals that dispersed (moved >4 m). We did not include individuals that moved less than 4 m in this analysis because the dispersal fate of these individuals is unknown, as dispersal could have occurred prior to our study or may occur in the future, thereby increasing morphological variation in these putative “nondispersers.” Model selection was based on Akaike information criterion (AIC). Our initial model only included four predictor variables that were based on a priori hypotheses of how morphology affects dispersal; therefore, we assumed low family‐wise error.

### 2.5 Performance assays

To test for a locomotion‐based trade‐off with dispersal distance, we assessed burst‐swimming performance in controlled experiments. We constructed an in‐stream chamber (71 cm long × 22.5 wide cm × 25 cm tall) that was placed in a pool in the stream channel in Zigzag brook so that salamanders did not experience any flow or incline during the swimming trials. The water depth in the chamber was 8–10 cm. Previously marked individuals captured in 2014 and 2015 underwent swimming trials. Salamanders were allowed to rest in cool stream water for 16–24 hr before undergoing swimming trials to minimize exhaustion and stress following capture. Salamanders were prodded a maximum of three times to elicit a swim response. Using dorsal‐view video, we sampled swimming trials at 60 frames per second using a GoPro Black 3+. We used a wide‐view to capture the length of the swimming chamber, which created distortion that we removed before kinematic analyses. We calculated an undistortion transformation using a gridded image and X‐ray of Moving Morphology (XROMM) Undistorter, and we applied the undistortion correction to each video file using the XrayProject 2.2.5 script in MATLAB (Brainerd et al., 2010). A contrasting bead attached with a rubber band on the salamander's torso served as an anatomical landmark, and this point was digitized in each frame in MATLAB using a custom script, DLTdv5 (Hedrick, 2008). We used Igor Pro (v.6) to derive peak velocity (m/s) and peak acceleration (m/s^2^) from digitized position data (m). These measures were obtained by averaging over a series of 11 digitized frames to minimize effects of random digitizing error that were inflated by taking derivatives. This smoothing may produce different values from instantaneous measures achieved with higher frame rates or from other averaging algorithms (Walker, 1998). However, the performance of all animals in this study was evaluated using the same methods, such that performance measures within this study are directly comparable. Salamanders were immediately returned to their last capture location following swimming trials.

The challenge of collecting both dispersal and performance data from the same set of individuals prevented us from assessing the repeatability of swimming performance, but other studies have demonstrated high repeatability of locomotor performance in amphibians (Kolok, 1999; Marvin, 2003; Walton, 1988). Additionally, the animals tested in this study were a part of a larger multiyear study and we chose to quantify swimming performance from a single trial per individual to reduce any effects of handling stress and fatigue on movement behavior postrelease (Langkilde & Shine, 2006). Our study therefore represents a distinct departure from the more common approach of collecting repeated performance measures from a single individual to quantify individual variation in maximal performance (e.g., Marras, Claireaux, McKenzie, & Nelson, 2010; Reidy, Kerr, & Nelson, 2000). We do not quantify maximal performance, but instead use a single measure per individual to quantify variation in performance across individuals.

To assess whether the same morphological variable(s) associated with dispersal distance also influenced swimming performance, we used stepwise multiple regression analysis to identify the most predictive model of each performance metric from the set of size‐adjusted trunk and limb variables (PC2s). This analysis was conducted for the same individuals for which we tested for relationships between morphology and dispersal distance (i.e., individuals that moved >4 m). We used linear regression to evaluate the possibility that swimming performance itself predicts dispersal distance, as many studies use proximate aspects of locomotor performance to infer dispersal ability (e.g., Arnold et al., 2016; Bringloe et al., 2013; Cormont et al., 2011). All statistical analyses were conducted in the program R version 3.3.1 (R Development CoreTeam, 2016).

## 3 RESULTS

### 3.1 Surveys

**Figure 2:**
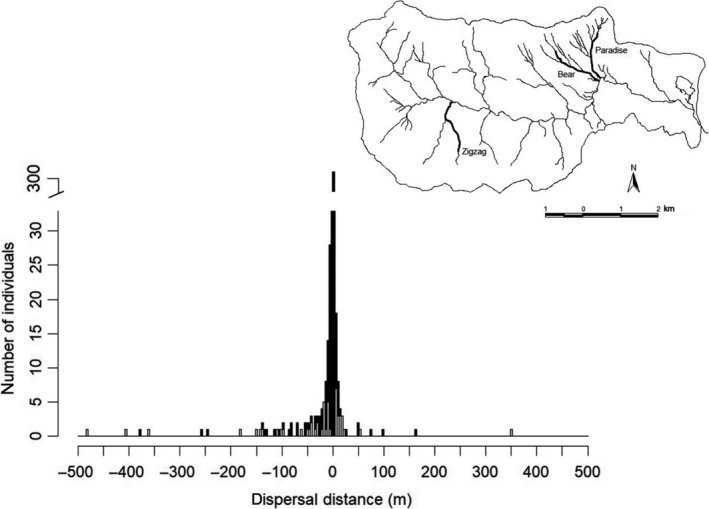
Dispersal distances of *Gyrinophilus porphyriticus* from three streams in the Hubbard Brook Watershed in central New Hampshire (inset map). Distances are from individuals recaptured between 2012 and 2015 (*n* = 575). Negative distances indicate downstream movements; positive distances indicate upstream movements. Data are binned in 4 m increments. Gray portions of the columns are individuals for which both morphological and performance data were collected (*n* = 50)

We marked 2,368 *G. porphyriticus* individuals over the 4‐year study period in the three study streams. Of these, 575 individuals were recaptured, including 159 adults and 417 larvae. There was no difference in the dispersal distributions of larvae and adults (Kolmogorov–Smirnov test, *p* > .28). Dispersal occurred in both downstream and upstream directions (Figure 2), and the mean of absolute dispersal distances (±1 *SD*) was 12.33 m ± 45.08. One hundred and thirty two individuals dispersed >4 m in either direction from their initial locations. The maximum dispersal distance detected was 481 m (Figure 2). Dispersal distances were negatively skewed (skewness = −1.75, where negative skewness represents downstream bias; Figure 2).

To test for locomotion‐based trade‐offs with dispersal distance, we needed individuals that dispersed in the field (moved >4 m) and had measures of swimming performance (*n* = 50). The mean of absolute dispersal distances (±1 *SD*) of this subset was 49.95 m ± 83.96, and included 26 adults and 24 larvae. The range of dispersal distances in this reduced dataset matched that of the full dataset, and the distributions did not differ (Kolmogorov–Smirnov test, *p* = .95; Figure 2).

### 3.2 Morphological variation

**Figure 3:**
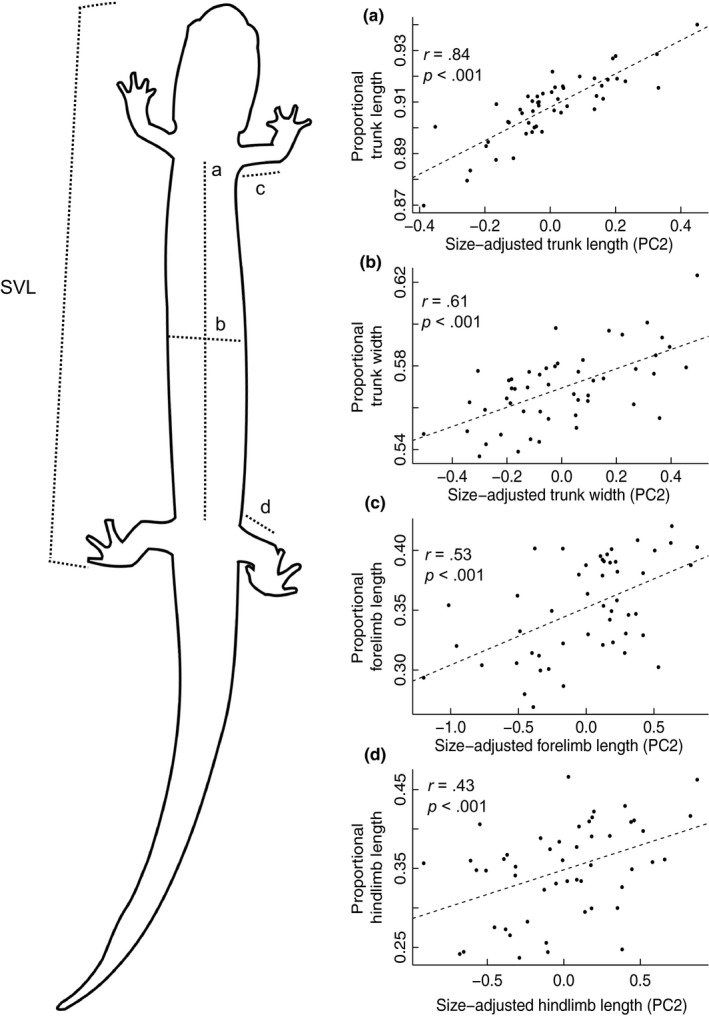
Correlations between size‐adjusted morphological variables (PC2 scores) and proportional size of each body element (e.g., log trunk length/log snout–vent length [SVL]) for *Gyrinophilus porphyriticus* individuals in the Hubbard Brook Watershed (*n* = 50). Letters in the top left of plots indicate corresponding measurements of (a) trunk length, (b) trunk width, (c) forelimb length, and (d) hindlimb length on salamanders. PC2 scores were from principal component analyses including each body measurement and SVL. The percentage of variation accounted for by these PC2s is indicated within each plot. Lines of best fit are plotted for each correlation to show trends

The first principal components of each of the four covariance matrices representing the four body elements were positively correlated with log‐transformed SVL, confirming that PC1s represented the generalized size of salamanders (*r* = .95–.99). The second principal components, therefore, represented size‐adjusted shape variables. Second principal components were positively weighted by the body measurements; therefore, the proportional size of each body element (e.g., log trunk length/log SVL) was positively correlated with PC2 score (*r* = .43–.84, *p* < .001, Figure 3) Among the PC2 values, only trunk length PC2 and trunk width PC2 were correlated (*r* = .58, *n* = 50, *p < *.001).

### 3.3 Dispersal distance

**Figure 4:**
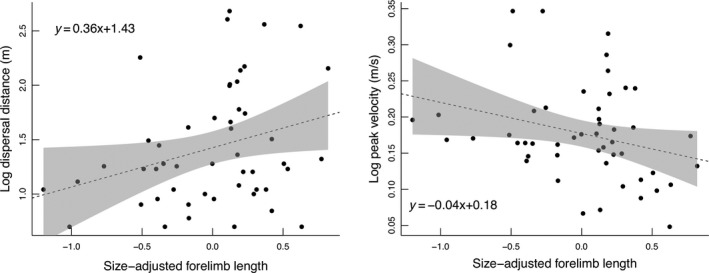
The relationship between sized‐adjusted forelimb length (PC2) and dispersal distance (left) and swimming velocity (right) in *Gyrinophilus porphyriticus* individuals that dispersed >4 m in the Hubbard Brook Watershed (*n* = 50). Dotted linear regression lines indicate significant associations (*p* < .05); gray bands indicate 95% confidence intervals. Size‐adjusted forelimb length is positively weighted by humerus length; therefore, individuals with longer forelimbs dispersed the farthest but swam at the lowest velocities

The best model of dispersal distance (log‐transformed) among dispersers (*n* = 50) included forelimb PC2 alone and received over twice as much support as the second‐ranked model, which included trunk width PC2 and forelimb PC2 (Table 1). Individuals with longer forelimbs dispersed farther (*β* = .36, *SE* = 0.17, *t* = 2.14, *p* = .037, *r* ^2^ = .07; Figure 4). Dispersal distance was unrelated to SVL and trunk and limb PC1s (*r* = .0–.1, *n* = 50, *p* = .49–.99), indicating that there was no ontogenetic variation in dispersal distance.

**Table 1:** Models of dispersal distance in *Gyrinophilus porphyriticus* larvae and adults in Bear, Paradise, and Zigzag brooks

Model	AIC	∆AIC	Likelihood	Weight
Distance ~ forelimb PC2	−61.97	0.00	1.00	0.56
Distance ~ trunk width PC2 + forelimb PC2	−60.38	1.59	0.45	0.25
Distance ~ trunk width PC2 + trunk length PC2 + forelimb PC2	−59.17	2.80	0.25	0.14
Distance ~ trunk width PC2 + trunk length PC2 + hindlimb PC2 + forelimb PC2	−57.26	4.71	0.09	0.05

### 3.4 Swimming performance

Log‐transformed peak velocity (mean: 0.18 m/s; range: 0.05–0.35 m/s) and log‐transformed peak acceleration (mean: 0.76 m/s^2^; range: 0.25–1.26 m/s^2^) were positively correlated (*r* = .82, *n* = 50, *p* < .001); therefore, we used only peak velocity as our swimming performance metric. The best model of peak velocity among dispersers included forelimb PC2 alone and received 1.7 times more support than the next best model, which included hindlimb PC2 and forelimb PC2 (Table 2). Individuals with shorter forelimbs attained the highest peak velocities (*β* = −.04, *SE* = 0.02, *t *= −2.06, *p* = .042, *r* ^2^ = .06; Figure 4). Peak velocity was unrelated to SVL and trunk and limb PC1s (*r* = .06–.15, *n* = 50, *p* = .31–.68), indicating that there was no ontogenetic variation in swimming velocity. Peak velocity was unrelated to dispersal distance (*β* = −.48, *SE* = 1.18, *t* = −0.41, *p* = .67).

**Table 2:** Models of peak swimming velocity in *Gyrinophilus porphyriticus* larvae and adults in Bear, Paradise, and Zigzag brooks

Model	AIC	∆AIC	Likelihood	Weight
Peak velocity ~ forelimb PC2	−272.38	0.00	1.00	0.50
Peak velocity ~ hindlimb PC2 + forelimb PC2	−271.29	1.09	0.58	0.29
Peak velocity ~ trunk width PC2 + hindlimb PC2 + forelimb PC2	−269.92	2.46	0.29	0.15
Peak velocity ~ trunk width PC2 + trunk length PC2 + hindlimb PC2 + forelimb PC2	−268.42	3.96	0.14	0.07

## 4 DISCUSSION

Our study is novel in demonstrating a trade‐off associated with continuous variation in dispersal distance and, specifically, that a phenotypic attribute associated with increased dispersal distances restricts another locomotor performance. These results provide empirical insight on the causes of variation in dispersal distance across individuals and constraints on the evolution of dispersal (Bonte & Dahirel, 2017; Burgess et al., 2015; Burton, Phillips, & Travis, 2010), and support an alternative to the historical view that dispersal distance is controlled by extrinsic environmental factors. As importantly, by integrating field and experimental data, this study shows the risk of relying on proximate measures of locomotor performance (e.g., swimming velocity) as proxies for dispersal ability (Arnold et al., 2016; Bringloe et al., 2013; Cormont et al., 2011). Our results suggest that these proximate performance measures may not only misrepresent dispersal ability, but instead reflect fundamental constraints on dispersal ability.

The positive relationship between forelimb length and dispersal distance suggests that *G. porphyriticus* individuals disperse primarily via walking—either underwater (larvae and adults) or overland (adults only)—because salamanders do not actively use their limbs for swimming (Delvolvé, Bem, & Cabelguen, 1997). This finding adds to a growing body of work linking limb morphology to dispersal or movement capacity (Arnold et al., 2016; Lowe & McPeek, 2012; Phillips, Brown, Webb, & Shine, 2006). Mechanistically, longer limbs increase stride length during terrestrial locomotion and allow the animal to move a greater distance per step, thereby lowering the cost of transport (Pontzer, 2007). There are few studies examining the role of limbs in underwater walking, but Ashley‐Ross et al. (2009) showed that limb kinematics of the California newt (*Taricha torosa*) are strikingly similar between aquatic and terrestrial environments, suggesting benefits of longer limbs for dispersal may be consistent across environments. Direct assessment of limb length and associated effects on costs of transport during underwater walking is needed to evaluate this possibility, as well as to evaluate the availability of walking as a dispersal mode for larval *G. porphyrititicus*, which are constrained to waterways. The absence of a relationship between hindlimb length and dispersal distance in our data may be a function of the reduced requirement for stability in aqueous environments, in contrast to walking on land where legs play a larger role in supporting the body (Ashley‐Ross, 1994).

Longer limbs increase hydrodynamic drag during swimming, which may explain why swimming velocity declined with forelimb length (Figure 4). Aquatic salamanders generally hold their limbs close to the body during swimming to reduce drag (Bennett, Simons, & Brainerd, 2001; Delvolvé et al., 1997). However, we noticed that *G. porphyriticus* individuals displayed a wide range of limb postures while swimming—in some cases extending them to be nearly perpendicular to the long axis of the body. We modeled drag as a function of forelimb length in *G. porphyriticus* and found that the longest‐limbed individuals could experience up to 18% more drag than the shortest‐limbed individuals (range 5.0–5.9 milliNewtons; see Appendix 1 for details). This increase in drag solely due to longer forelimbs could represent a significant selective pressure on limb length in aquatic salamanders. Measures of maximal performance, however, are needed to thoroughly evaluate the role of natural selection on driving variation in limb length and associated swimming performance, as maximal performance is expected to be most closely tied to fitness (Irschick, Meyers, Husak, & Galliard, 2008).

Forelimb drag could be problematic for both burst and sustained swimming, strengthening our hypothesis that the positive association we report between dispersal distance and limb length stems from improvements to walking, rather than swimming, performance. However, increased drag experienced by long‐limbed individuals could also function to increase dispersal distances. In aquatic organisms, body morphology can influence downstream displacement by directional water flows, with some body shapes better able to hold station against oncoming flows (Blake, 2006; Webb, Gerstner, & Minton, 1996). It is possible that higher drag experienced by long‐limbed salamanders may increase the energetic cost of resisting flow and result in greater distances displaced downstream relative to short‐limbed salamanders. This mode of dispersal represents a simple alternative hypothesis to the functional role of limb length in terrestrial or aquatic walking for dispersal, but is only relevant for downstream dispersal.

Our finding that the same trait was linked to both dispersal distance and swimming performance, but in opposite ways, is indicative of an adaptive trade‐off. Using proximate performance measures, trade‐offs between endurance and speed have been shown in other species (Bennett, Garland, & Else, 1989; Reidy et al., 2000), and our results may reflect a similar relationship. We did not measure endurance directly, but our results suggest that dispersal distance may be determined by the reduction in transport costs of walking with increased stride length or the passive use of drag, rather than by improvements in swimming performance. Swimming speed has, however, been linked to predator escape in larval amphibians (Dayton, Saenz, Baum, Langerhans, & DeWitt, 2005), including larval salamanders (Storfer, 1999), and both adult and larval *G. porphyrticus* are susceptible to predation (Brodie et al., 1979; Resetarits, 1991, 1995). Therefore, it is likely that predation pressure represents a strong selective force shaping swimming performance in this system.

The lack of correlation between body size and dispersal distance is surprising because other ecological interactions change with body size in *G. porphyriticus*. For example, predation pressure from brook trout is size‐dependent, with larvae being more affected than adults due to the gape limitation of brook trout (Lowe, Nislow, & Bolger, 2004; Resetarits, 1995). Thus, if dispersal were extrinsically controlled by environmental factors (Carlquist, 1981; Morales, 2002; Tufto et al., 1997), we would expect that dispersal distance might also change with body size and life‐history stage. Because we did not detect these ontogenetic relationships, we interpret our findings as support for the role of natural selection in maintaining variation in dispersal phenotypes and distances, rather than dispersal distance being conditional on stage/size or environmental cues. Tests of the fitness consequences and genetic basis of the forelimb phenotype are clearly needed to definitively assess this interpretation. Furthermore, given the complexity of the dispersal process (Nathan, 2001; Ronce, 2007), and the scatter in our data (Figure 4), accurate predictions of dispersal distance will likely rely on models that incorporate both individual traits and extrinsic environmental factors (Bocedi, Zurell, Reineking, & Travis, 2014; Henry, Coulon, & Travis, 2016).

## CONFLICT OF INTEREST

None declared.

## AUTHOR CONTRIBUTIONS

B.R.A, J.M.D, and W.H.L conceived the ideas and designed the methodology; B.R.A and J.M.D collected field data; B.R.A collected experimental data; B.R.A, B.W.T, and W.H.L analyzed the data; B.R.A led the writing of the manuscript; and all authors contributed feedback to the drafts and gave the final approval for publication.

## Data Availability

Individual dispersal, morphological, and performance data can be found on Dryad Digital Repository: https://doi.org/10.5061/dryad/nk6b116.

## Appendix 1

### Drag calculation

To explore the potential for forearm length to increase drag, we calculated the extent to which the range of forearm lengths represented in our study salamanders might increase drag according to the equation

$$F_{D}=\frac{1}{2}\rho \upsilon^{2}C_{D}A$$

where *F*_D_ is the force of drag, ρ is the density of water (1,000 kg/m^3^), *υ* is the velocity of water relative to the salamander, *C*_D_ is the coefficient of drag, and *A* is the cross‐sectional area perpendicular to the flow. We held velocity constant at 0.29 m/s, the mean swimming velocity of salamanders in this study. The coefficient of drag for a cylinder at Reynolds numbers ranging from 10^2^ to 10^5^ is one, which we considered reasonable for salamanders in headwater stream environments. For cross‐sectional area, we simplified the shape of the salamander to a circle (trunk) with two rectangles (forelimbs) to represent the widest part of the salamander with forelimbs perpendicular to flow. The circular area was calculated from the average trunk width of the 50 dispersers (11.38 mm). The rectangular area of the limbs was calculated based on an average width of 3 mm and the length varied according to the humerus length measured from each photograph (mean = 4.59 mm). Thus, the only term that varied in the drag calculations was cross‐sectional area, as a function of variation in forelimb length.
